# Ocular surface chemical injury treated by regenerating agent (RGTA, Cacicol20)

**DOI:** 10.3205/oc000079

**Published:** 2017-10-25

**Authors:** Melih Ustaoglu, Nilgun Solmaz, Feyza Onder

**Affiliations:** 1 Sisli Hamidiye Etfal Training and Research Hospital, Ophthalmology Clinic, Istanbul, Turkey; 2 Haseki Training and Research Hospital, Ophthalmology Clinic, Istanbul, Turkey

**Keywords:** ocular surface chemical injury, chemical burns, regenerating agents, RGTA, Cacicol20

## Abstract

**Objective:** To present the successful outcome of regenerating agent (RGTA) treatment in a patient with severe ocular surface chemical injury.

**Methods:** Case report

**Results:** A 14-year-old female patient was admitted to our clinic following chemical burn in the left eye. Her best corrected visual acuity (BCVA) was 20/40; and she had total corneal and 75% conjunctival epithelial loss, corneal haze, and limbal ischemia for nine clock hours in the left eye. The patient had already received standard therapy consisting of patching, preservative-free artificial tears, topical netilmicin, topical dexamethasone, oral doxycycline, and vitamin C for two weeks. We initially cleaned the conjunctival necrotic tissues, applied the silicon hydrogel bandage contact lens, exchanged the topical netilmicin with preservative-free moxifloxacin and supplemented this therapy with RGTA (Cacicol20, Paris, France) once in two days. The BCVA of the patient improved to 20/20 and the ocular surface re-epithelization was completed on day 20.

**Conclusion:** RGTAs are effective biological agents for the treatment of corneal epithelial defects following severe ocular surface chemical injuries.

## Introduction

Ocular surface chemical injuries are one of the most serious ocular emergencies which may cause a range of complications from corneal epithelium defects to corneal perforation [1]. Chemical substances cause destruction of cellular components, denaturation and degradation of collagenous tissues, and release of inflammatory mediators by hydrolysis of intracellular and extracellular proteins. Immediate and intensive treatment is required for the recovery of the ocular surface following the chemical injury. 

Regenerating agents (RGTAs) are biopolymers developed as heparan sulfate analogues resistant to enzymatic degradation. They take the place of degraded heparin sulfates in injured tissue and bind to extracellular matrix (ECM) proteins and growth factors; they protect ECM from proteolysis and reconstruct the micro-medium necessary for wound healing with inhibition of various proteolytic enzymes, controlling of inflammation, and regulation of collagen synthesis [2]. 

We present here the successful outcome of RGTA (Cacicol20, Paris, France) treatment in a patient with severe ocular surface chemical injury.

## Case description

**Figure 1:**
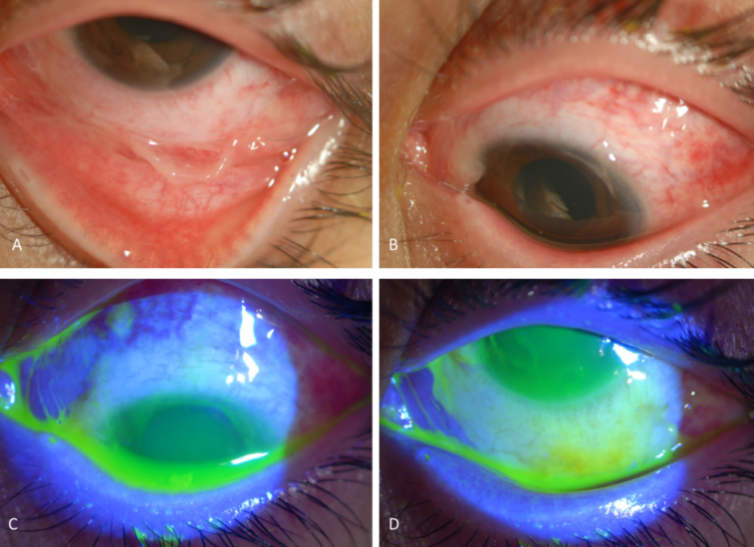
Total corneal and 75% conjunctival epithelial loss and limbal ischemia for nine clock hours, two weeks after the chemical injury

A 14-year-old female patient was admitted to our clinic following a chemical injury of the left eye. She stated that a 200 ml bottle of rose cologne had exploded in her hand and a large amount of the cologne had spread into the left eye. She had already received standard therapy consisting of patching, preservative-free artificial tears six times a day, topical netilmicin four times a day, topical dexamethasone six times a day, oral doxycycline 100 mg per day, and vitamin C 1000 mg per day for two weeks. On ophthalmic examination, her right eye was unremarkable. The best corrected visual acuity (BCVA) of the left eye was 20/40; slit lamp examination revealed total corneal and 75% conjunctival epithelial loss, corneal haze, and limbal ischemia for nine clock hours (Figure 1 (Fig. 1)). The patient was diagnosed with grade IV ocular surface damage on the basis of Dua classification [3]. After cleaning the conjunctival necrotic tissues, a silicon hydrogel bandage contact lens was applied to the ocular surface and topical netilmicin was exchanged with preservative-free moxifloxacin. In addition, this therapy was supplemented with topical RGTA (Cacicol20) once every two days.

**Figure 2:**
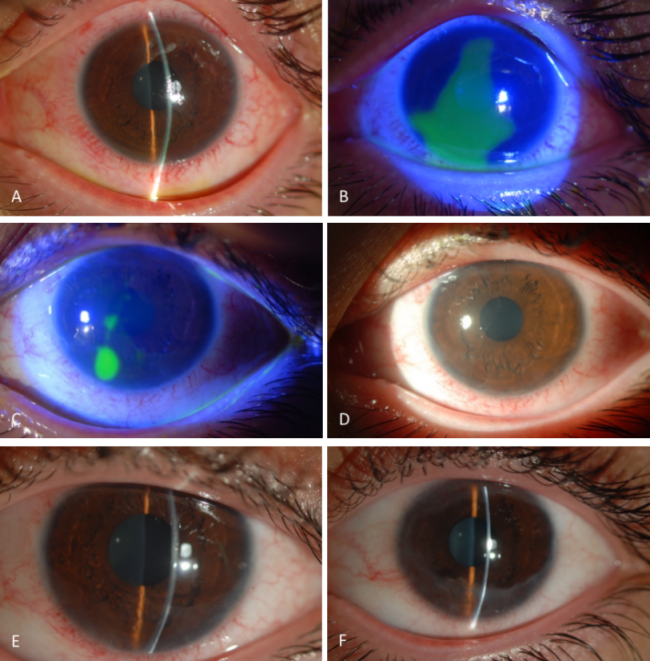
(A,B): Total conjunctival and two third of the corneal epithelial healing, a week after the RGTA treatment. (C,D): Significant reduction in the size of the epithelial defect, at the end of the second week. (E): Complete healing on the corneal surface with mild irregularity of the peripheral corneal epithelium, on day 20. (F): A month after the complete healing, approximately 300º conjunctivalization is seen at the peripheral cornea.

Following the RGTA treatment, the conjunctival epithelial defects started to shrink and ocular irritation signs reduced rapidly. Within the first week of the treatment, the total conjunctival surface and two thirds of the corneal epithelium healed (Figure 2A,B (Fig. 2)). Following the first week, the topical dexamethasone dose was reduced twice a day and the corneal epithelial defect shrank to a size of 1.5x2.0 mm (Figure 2C,D (Fig. 2)) at the end of the second week. On day 20, the corneal re-epithelialization was completed totally (Figure 2E (Fig. 2)) and the BCVA increased to 20/20. The RGTA treatment was discontinued after complete healing of the corneal epithelial defect. During this period, no irritation signs or side effects were observed. A week after the corneal re-epithelialization had been completed, conjunctivalization started from superior and inferior limbus to the corneal surface. Within a month, approximately 300 degree conjunctivalization occurred on the peripheral cornea (Figure 2F (Fig. 2)). The treatment including artificial tears, topical loteprednol etabonate 0.5% three times a day (tapered after two months), and topical cyclosporine 0.05% four times a day was continued during the following 8 months. The conjunctivalization slightly regressed and the visual acuity remained stable in this period.

## Discussion

Ocular surface chemical injuries are vision-threatening serious ocular emergencies. The treatment strategies for chemical injuries should be based on the severity of the disease. Preservative-free topical antibiotics, steroids and artificial tears, and cycloplegic agents are mostly sufficient for patients with mild ocular surface chemical injuries, whereas additional treatments are required for the severe burns. Ascorbic acids can be used as topical or systemic therapy to stimulate collagen synthesis. Doxycycline reduces the inflammation by inhibiting matrix metalloproteinases (MMPs) and contributes to the corneal re-epithelization. Autologous platelet-rich plasma also promotes corneal re-epithelization and reduces inflammation by content of growth factors and anti-inflammatory agents on severe chemical corneal burns [4].

Amniotic membrane transplantation (AMT) is a successful surgical treatment of severe burns in the acute period. It generates the physical and molecular effects. Closure of the burned ocular surface with amniotic membrane reduces pain and inflammation and also releases the molecular agents as epidermal growth factor (EGF) and transforming growth factor, beta-1 (TGFB1) that have a role in wound healing [5]. Limbal stem cell transplantation (LSCT) is another effective surgical procedure, which reduces the corneal vascularity and opacity, and increases the corneal re-epithelization in severe limbal ischemia.

RGTAs are new biological agents that influence corneal epithelization by inhibiting proteolytic enzymes, stimulating growth factors, and regulating collagen synthesis. Although clinical use is currently limited, some reports promise successful outcomes of RGTA in treatment of chronic corneal ulcers, neurotrophic ulcers [6], and persistent epithelial defects [7] resistant to conventional treatments. As far as we know, there is no publication in the medical literature reporting the use of RGTA for ocular surface chemical injury in humans. However, Cejkova et al. [8] showed that RGTAs decrease the corneal inflammation and neovascularization and improve the corneal re-epithelialization at rabbit corneas injured by alkali chemicals compared to placebo. 

In our patient, there was a severe corneal and conjunctival chemical burn. Although the AMT, LSCT are successful surgical treatments in ocular surface chemical injuries, the topical RGTA treatment was preferred because it is a nonsurgical therapeutic approach. The RGTA treatment reduced the inflammation and promoted the corneal re-epithelization in our patient. However, the success of RGTAs in the treatment of ocular surface chemical injuries should be evaluated by prospective controlled trials.

## Conclusion

RGTAs are promising biological agents which are successful in the treatment of severe corneal epithelial defects. These agents may be used in supplement to standard therapy or alternative to surgical treatments such as AMT and LSCT for severe ocular surface chemical injuries. 

## Notes

### Competing interests

The authors declare that they have no competing interests.
